# Characterization of the Nutraceutical Quality and Antioxidant Activity in Bell Pepper in Response to Grafting

**DOI:** 10.3390/molecules181215689

**Published:** 2013-12-16

**Authors:** Celia Chávez-Mendoza, Esteban Sánchez, Elizabeth Carvajal-Millán, Ezequiel Muñoz-Márquez, Alexandro Guevara-Aguilar

**Affiliations:** 1 Coordinación en Tecnología de Productos Hortofrutícolas y Lácteos Centro de Investigación en Alimentación y Desarrollo A C. Avenida Cuarta Sur No. 3820 Fraccionamiento Vencedores del Desierto. Cd. Delicias, Chihuahua C.P. 33089, Mexico; E-Mails: celia.chavez@ciad.mx (C.C.-M.); emunoz@ciad.mx (E.M.-M.); aguevara@ciad.mx (A.G.-A.); 2 Coordinación de Tecnología de Alimentos de Origen Animal. Centro de Investigación en Alimentación y Desarrollo, A.C. Carretera a la Victoria Km. 0.6 AP 1735. Hermosillo, Sonora C.P. 83304, Mexico; E-Mail: ecarvajal@ciad.mx

**Keywords:** rootstock, bioactive compounds, *Capsicum annuum* L.

## Abstract

The grafting of fruits and vegetables influences fruit quality. The aim of the present work was to assess the effect of the rootstock and the scion on the antioxidant activity and the content in vitamin C, total phenols, lycopene and β-carotene of bell pepper. The cultivars Fascinato and Jeanette were used as scion and Terrano was used as rootstock. Four harvests in the production cycle of the vegetable were analyzed in a cultivation system under shading nets. The results indicate statistical differences in the content of these bioactive compounds between the varieties, between grafting and not grafting and between sampling dates (*p* ≤ 0.05). The vitamin C content, β-carotene, and antioxidant capacity proved significantly higher in Fascinato than in Janette. On average, grafting increased β-carotene and vitamin C concentrations and improved the antioxidant capacity, but had no influence on the total phenol or lycopene contents. It is concluded that grafting to the rootstock Terrano improves the nutritional quality of the fruit produced in both varieties of bell pepper studied.

## 1. Introduction

Bioactive compounds are essential and non-essential substances found in Nature as part of the food chain and are known to influence human health [1]. Found in small quantities in different foods, these affect different activities at the physiological or cellular level [2]. One such food, the bell pepper, has a high content in vitamin C, carotenes, phenols, capsaicinoids, xanthophylls, and flavonoids, in addition to having high antioxidant activity [3,4]. These micronutrients give the bell pepper qualities that have led to its use in traditional medicine against degenerative diseases, intestinal disorders, dysentery [5], and different problems related to mental health [6], among other ailments.

The content in bioactive compounds in bell pepper depends on many factors, such as variety, cultivation conditions, the degree of ripeness at harvest, and post-harvest handling [3,6]. It has been reported that the variety of red pepper has greater antioxidant power due to the higher content in oxygenated carotenoids, such as capsanthin, capsorubin, and cryptocapsin [7]. These and other antioxidants have ample power to neutralize free radicals in the cells, which can adversely affect lipids, proteins, and cell DNA, causing diseases in the human such as cell ageing, mutagenesis, and carcinogenesis [8].

According to epidemiological evidence, the consumption of foods rich in antioxidant nutrients strongly correlates with low morbidity and mortality in humans, and thus dietary chemo-prevention has emerged as an economical way to control chronic diseases such as cancer [9].

Therefore, research is currently being dedicated to implementing technologies that not only augment food-crop production but also improve nutritional quality, with an emphasis on bioactive compounds. One such technology is the grafting of crops [10], a propagation method consisting of joining the aerial part of one plant (scion) to the stock of another that is already rooted (rootstock), resulting in an autonomous individual formed by two plants [11].

Nowadays, vegetable grafting is considered an innovative technique and is in increasing demand by farmers. This technique has been applied in fruits and vegetables for diverse purposes, such as disease resistance, tolerance to extreme temperatures, salinity, and drought; improved water and nutrient uptake; increased plant vigor; lengthening of the harvest period [12]; and stronger performance and tolerance under environmental stress [13].

In addition, this technology provides advantages against abiotic stress, reducing the need for chemical or fertilizer applications, improving fruit quality [11] and promoting the production of specific phytochemical compounds [14].

Bell pepper has rarely been grafted because this plant is highly resistant to soil diseases and environmental stress, but currently this practice is increasing in bell pepper production systems due to the elimination of methyl bromide as a soil fumigant and the rise of new pathogens and bacterial diseases [15]. However, in the world in general, the use of grafting is not as widespread in bell pepper plants as it is in other vegetable fruit species. As a consequence, there has been little discussion about grafting behaviour in the nursery, compatibility between rootstock and scion, grafted plant development, tolerance to biotic and abiotic stresses, potential commercial cultivar yield and quality, *etc.* In addition, very little attention has been paid to how the use of different rootstocks can affect fruit quality in grafted sweet pepper [16].

The main applications of grafting in bell pepper have been to control nematodes, soil diseases [17,18] and wilt caused by *Phytophtora capsici* [19,20,21]. The effect of this practice on the morphological characteristics of the fruit have also been evaluated [22], but few studies examine grafting in relation to the nutritional composition of bell peppers or indicate how the technique influences this composition, especially regarding antioxidant activity and content in bioactive compounds.

The aim of the present work was to assess the effect of the rootstock and the scion on the antioxidant activity and the content in bioactive compounds of bell pepper (*Capsicum annuum* L.).

## 2. Results and Discussion

### 2.1. Vitamin C

**Figure 1:**
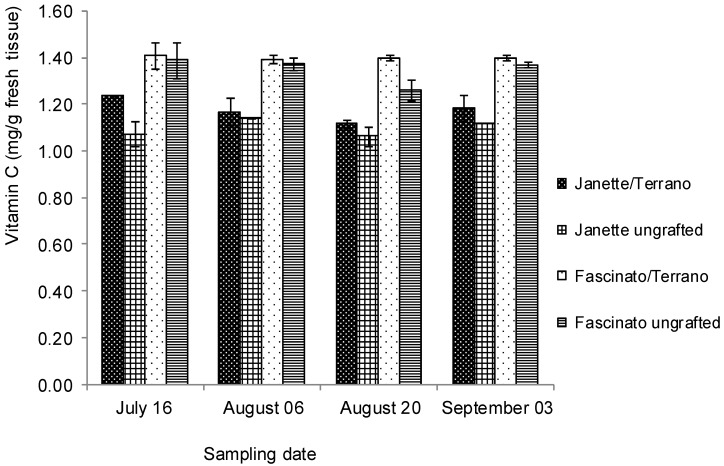
Vitamin C concentration in two bell pepper varieties grafted and ungrafted.

Fresh bell pepper is an excellent source of vitamin C. Data from other studies show that vitamin C levels in peppers can increase as the fruit ripens [3]. The vitamin C values of two commercial varieties of bell pepper, with and without grafting, on four sampling dates are presented in Figure 1.

In all samplings, the variety Fascinato had a greater vitamin C concentration than did Janette, (*p* ≤ 0.05), averaging 1.35 and 1.13 mg/g of this compound in fresh tissue, respectively. Also, on average, the grafted pepper had 3.9% more vitamin C than did the ungrafted one (*p* ≤ 0.05). The best treatment for this compound was Fascinato grafted (Fascinato/Terrano) in the first sampling, with 1.40 mg/g of fresh tissue, while its vitamin C concentration showed no significant differences over time. Similar results have been reported by Howard *et al.* [3] in ripe peppers of the variety Yellow Bell 47. In the present study the limit of detection and limit of quantification of the analytical method used for vitamin C quantification were 1.3 and 3.9 µg/mL, respectively.

In this work, grafted Fascinato had 1.69% more vitamin C with than ungrafted, whereas grafted Janette (Janette/Terrano) registered 6.51% more than when ungrafted. This finding agrees with the results reported by Proietti *et al.* [23] who stated that total vitamin C content for grafted mini-watermelon plants was higher by 7% than those from ungrafted plants. Similarly, Huang *et al.* [24] reported an increase of vitamin C contents in cucumber fruits harvested from plants grafted onto figleaf gourd and “Chaofeng Kangshengwang”. By contrast, Turhan *et al.* [25] found that vitamin C content in tomato was strongly reduced by grafting, and López-Marín *et al.* [16] found no significant differences in the vitamin C concentration in Herminio variety bell pepper between ungrafted individuals and those grafted onto Terrano or Creonte, although differences were found when Herminio was grafted onto Atlante, vitamin C increasing in concentration. Similarly, Gisbert *et al.* [20] found no differences in the vitamin C content in Coyote and Almuden bell peppers when grafted onto Foc and Charlot.

According to the present results, grafting increased the concentration of the vitamin C in the bell peppers, this effect being greater in Janette, indicating that this variety was compatible with the Terrano rootstock. In agreement with San Bautista *et al.* [26], the enhanced fruit quality is due to a strong connection between the scion and rootstock, facilitating the flow of water and nutrients, enhancing photosynthesis, and consequently improving the availability of carbohydrates as an energy source for the plant.

### 2.2. Total Phenols

**Figure 2:**
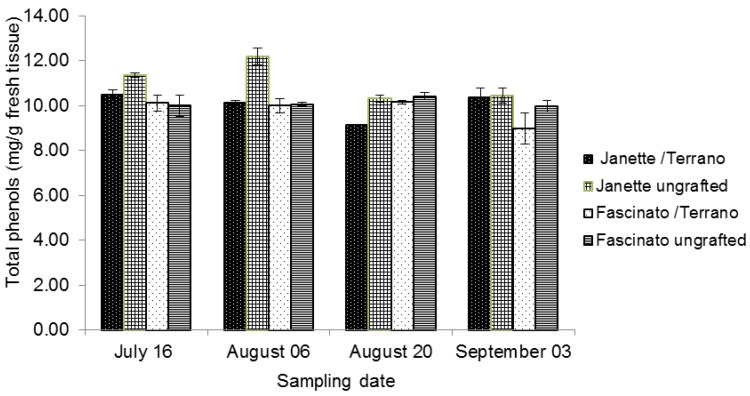
Total phenol concentrations in two bell pepper varieties grafted and ungrafted.

Phenol compounds are found in considerable quantities in many fruits and vegetables and thus form an integral part of the human diet. Their study has attracted great interest because their consumption is associated with reduced risks of cardiovascular disease and certain types of cancer [27]. In the present work, the total phenol content was determined in two commercial varieties of bell pepper, with and without grafting, on four sampling dates (Figure 2). Significant differences were found between varieties (*p* ≤ 0.05), as the variety Janette presented higher concentrations of total phenols than did Fascinato, averaging 10.54 and 9.95 mg/g of fresh tissue, respectively. These values were higher than found by Zhuang *et al.* [4] in red pepper *Capsicum annum L* harvested in Yunnan Province, China (with 2.1 mg/g of fresh tissue). However, these values were lower than reported by Blanco-Ríos *et al.* [28], who found levels of 12.89 and 12.9 mg/g of total phenols in fresh tissue for Mazurca variety red bell peppers and Taranto yellow bell peppers, respectively.

Bell pepper contains high concentrations of total phenols, which diminish as the fruit ripens [29]. Navarro *et al.* [30] reported a concentration of 5.47 mg/kg of dry weight in green peppers and 5.25 mg/kg in red peppers of the varieties Orlando and California. In the present work, the statistical analysis showed significant differences in the total phenol content between grafted and ungrafted peppers (*p* ≤ 0.05). On average, the total phenol concentration in ungrafted bell pepper was 6.36% greater than in grafted.

In addition, given the average found in the four samplings, the best treatment for this compound was ungrafted Janette, with 11.07 mg/g of fresh tissue, followed by ungrafted Fascinato, with 10.11 mg/g; then grafted Janette (10.02 mg/g) and finally grafted Fascinato (9.81 mg/g of fresh tissue). Similar results have been reported by López-Marín *et al.* [16], who found lower phenol values in grafted bell pepper (variety Herminio grafted onto Atlante, Creonte, and Terrano) than in the ungrafted varieties with and without shading. Vinkovic-Vrcek *et al.* [31] also found that the graft diminished the phenol concentration in tomato variety Tamaris grafted onto Heman, Efiato, and Maxifort. Similarly, Moncada *et al.* [32] reported that total phenolic content was greater in ungrafted eggplants than grafted.

In the present study, significant differences (*p* ≤ 0.05) were found between sampling dates. The highest concentration appeared in the second sampling, when the highest level of total phenols was registered for Janette without grafting (12.177 mg/g of fresh tissue). Under the experimental conditions used in the present study, the limit of detection and limit of quantification of the analytical method used for phenols quantification were 1.38 and 4.19 µg/mL, respectively.

### 2.3. Lycopene

**Figure 3:**
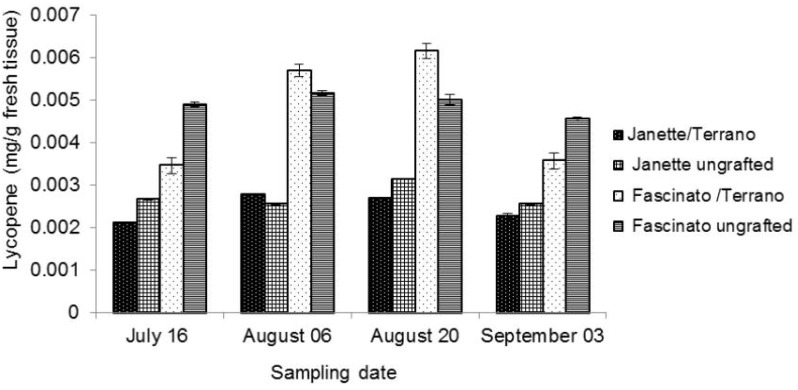
Lycopene concentration in two bell pepper varieties grafted and ungrafted.

Lycopene reportedly has antioxidant and anti-carcinogenic effects at the cellular level, and thus its consumption is considered to be beneficial to human health [33]. Bell pepper is a major source of this component [27]. In the present study, the lycopene concentration was determined in two commercial varieties of bell pepper, with and without grafting on four sampling dates (Figure 3). In all the samplings, higher lycopene concentrations (*p* ≤ 0.05) were found in the Fascinato variety than in Janette, averaging 0.0048 and 0.0025 mg/g of licopene in fresh tissue, respectively. These values were lower than reported by Navarro *et al.* [30] in Orlando and California pepper varieties, both with completely red fruits (0.322 mg/g) as well as half green and half red ones (0.138 mg/g of lycopene in fresh tissue).

In the present study the limit of detection and limit of quantification of the analytical method used for lycopene quantification were 0.01 and 0.02 µg/mL, respectively.

The statistical analysis indicated significant differences (*p* ≤ 0.05) in the quantity of lycopene in grafted and ungrafted bell peppers, the ungrafted peppers averaging 5.82% more lycopene than in grafted ones.

The variety-graft interaction affected the lycopene content (*p* ≤ 0.05). The best treatment was ungrafted Fascinato, which presented 3.75% more lycopene than when grafted. Meanwhile, ungrafted Janette had 9.55% more than when grafted, signifying that this technology did not stimulate this bioactive compound in these varieties. However, this could depend on the sampling date, as the lycopene content in grafted Fascinato in the second and third sampling was significantlly higher than in the first and fourth sampling (*p* ≤ 0.05).

Previous studies have documented that grafting affects the nutritive quality of vegetables. Davis and Perkins-Veazie [34] found that this technique boosted the lycopene concentration in watermelon, a result found also by Gerster [35] in tomato. In contrast, Mohammed *et al.* [36] found a decrease for lycopene in tomato cv Cecilia F1 grafted onto “Beaufort”, “He-Man” and local Syrian tomato roostock. Similary, Khah *et al.* [37] found no difference in lycopene content between ungrafted tomato cv. “Big Red” (*S. lycopersicum* L.) and grafted tomatoes onto “He-Man” and “Primavera” rootstocks under open-field and greenhouse conditions. In the present study, the increase in the concentration of lycopene by grafting was detected only in the Fascinato variety in the second and third sampling, as well as in Janette during the second sampling.

### 2.4. β-Carotene

**Figure 4:**
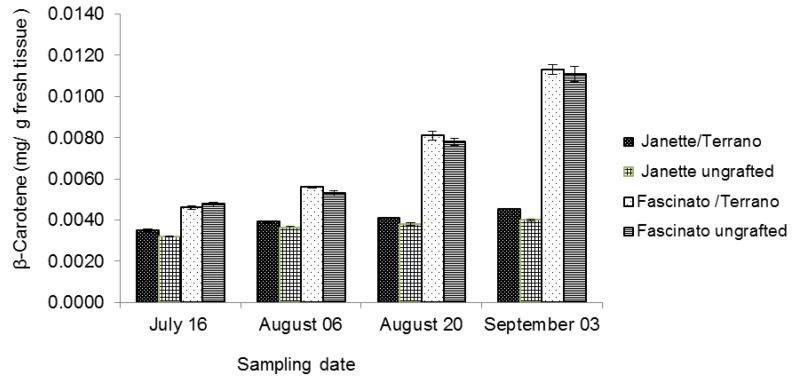
β-carotene concentration in two bell pepper varieties grafted and ungrafted.

A precursor of vitamin A, β-carotene reportedly may help protect against several types of cancer, especially lung, gastrointestinal, breast, and prostate [38]. Bell peppers are a good source of carotenes, which can vary in their composition and content due to different genotypes, cultivation, and state of ripeness [39,40]. The values of the β-carotene concentrations in the present study for the two commercial bell pepper varieties with and without grafting on four sampling dates are presented in Figure 4. In all the samplings, higher β-carotene concentrations were found for Fascinato than for Janette (*p* ≤ 0.05), averaging 0.0074 and 0.0039 mg/g of β-carotene in fresh tissue, respectively. The statistical analysis revealed significant differences between grafted and ungrafted peppers (*p* ≤ 0.05); on average, the grafted pepper had a 52% higher β-carotene concentration that when ungrafted. Similarly, Davis *et al.* [41], studying watermelon, reported 20% more total carotene after grafting. Fernandez-Garcia *et al.* [42] also, found large increase in β-carotene content due to grafting in tomato (cvs. “Fanny” and “Goldmar”) grafted onto hybrid AR-9704 tomato rootstock. Similary, Condurso *et al.* [43] reported an increase in β-carotene content in melon cv. Proteo grafted onto pumpkin hybrids rootstocks (namely Polifemo, AS10, P360 and ELSI).

Some authors have related the highest carotenoid content in grafted vegetable crops to the high K concentration in the fruit since this cation is important for protein synthesis and the activity of acetic thiokinase. This enzyme is involved in the formation of acetyl CoA, a molecule implied in biosynthesis of isopentenyl diphosphate, the first precursor of carotenoids. Nevertheless it can be believed that grafting increase carotenoid content of fruits also because of the higher amount of Mg in grafted fruits since many of the higher plant enzymes involved in the carotenoid biosynthesis require a divalent ion, typically Mg or Mn, for optimum activity [43].

In the present work, a considerable increase was found in the β-carotene content over the sampling period (*p* ≤ 0.05). Grafted Fascinato, which proved to be the best treatment, increased its initial value 2.5-fold, from the first to the fourth sampling. Furthermore, it had 54% more β-carotene than in grafted Janette. This latter variety did not register major changes β-carotene over the four samplings.

On average, the variety Fascinato with grafting had 0.0074 mg/g of fresh tissue, followed by Fascinato without grafting, with 0.0072 mg/g; then grafted Janette, with 0.0040 mg/g, and finally ungrafted Janette, with 0.0037 mg/g of fresh tissue. These values were far lower than those reported by Navarro *et al.* [30] in red peppers. While the β-carotene concentration of the Fascinato variety was similar to that found by Perucka and Materska [44] in King Arthur and Red Knight pepper varieties but higher than that reported by Blanco-Ríos *et al.* [28] in Mazurca red peppers harvested in northeastern Mexico (0.043 mg/g). The differences may be due to the intrinsic characteristics of each variety, cultivation conditions, and the state of ripeness, among other factors.

In the present study the limit of detection and limit of quantification of the analytical method used for β-carotene quantification were 0.03 and 0.10 µg/mL, respectively.

### 2.5. Antioxidant Capacity

**Figure 5:**
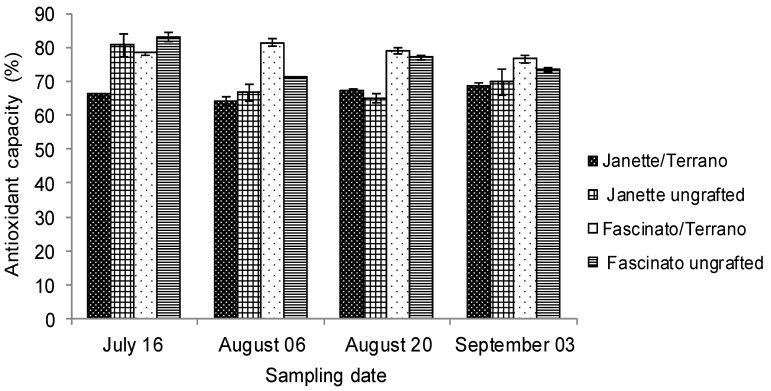
Antioxidant capacity of two bell pepper varieties grafted and ungrafted.

Antioxidants protect humans against free radicals, which can damage cells and increase the risk of developing cancer, cardiovascular diseases, and other degenerative disorders [45]. Figure 5 presents the antioxidant capacity of Fascinato and Janette variety bell peppers grafted with rootstock Terrano sampled on four different dates. The results show that Fascinato had greater antioxidant capacity than did Janette in all the samplings (*p* ≤ 0.05), averaging 77.53% and 68.48%, respectively. These values are far higher than those found by Ghasemnezhad *et al.* [29] in Arian, Marona, Zorro, Y-43-09, and Y-43-07 varieties cultivated hydroponically. Meanwhile, Blanco-Ríos *et al.* [28] also found that the red peppers (Mazurca) had greater reducing capacity than did orange peppers (Simpaty), yellow (Taranto), or green (Orion) varieties. In agreement with Sun *et al.* [46], red peppers in general exceed green ones in flavonoid content, which notably contributes to the antioxidant power of the fruit.

No significant statistical differences were found (*p* ≥ 0.05) in the average antioxidant capacity of grafted and ungrafted peppers, but there was a variety-graft interaction. Grafted Fascinato proved to be the best treatment (*p* ≤ 0.05), averaging 78.81% ± 1.96% of antioxidant capacity, followed by ungrafted Fascinato, with 76.26% ± 4.73%; then ungrafted Janette, with 70.54% ± 6.81%, and finally grafted Janette, with 66.43% ± 1.82% antioxidant capacity. These data indicate that grafting in Fascinato improved its antioxidant capacity, but not in Janette. Similarly, López Marín *et al.* [16] found no effect of grafting on the antioxidant capacity of bell pepper variety Herminio grafted onto Atlante, Creonte, and Terrano. Similar results were reported by Qaryouti *et al.* [47] who found that the antioxidant capacity, was reduced or slightly improved by grafting in tomato cv Cecilia F1 grafted onto “He-Man” and “Spirit”.

In terms of antioxidant capacity, Fascinato registered a greater concentration of β-carotene, lycopene, and vitamin C than did Janette. In addition, the results reveal an inverse correlation (*p* ≤ 0.05) of the antioxidant capacity with β-carotene (r = −0.61) as well as a direct correlation with total phenols (r = 0.59) and lycopene (r = 0.66) in grafted Fascinato, while no such correlations were found in Janette (*p* ≥ 0.05).

The statistical analysis showed significant differences between sampling dates (*p* ≤ 0.05). Grafted Fascinato proved to have greater antioxidant capacity during the second and third sampling, coinciding with the concentration of lycopene in the fruits on these two sampling dates. In the present study the limit of detection and limit of quantification of the analytical method used for antioxidant capacity determination were 0.61 and 1.85%, respectively.

## 3. Experimental

### 3.1. Handling and Experimental Design

In the present study, grafted and ungrafted bell pepper fruits were analyzed to determine the content in bioactive compounds. The pepper fruits were obtained from a commercial crop belonging to an agricultural company and grown under net shading.

For the experiment, the commercial varieties of bell pepper (Syngenta Seed, Houston, TX, USA) Fascinato (red fruits) and Janette (yellow fruits) were used as scion and grafted on the commercial rootstock Terrano (Syngenta Seeds) due to its resistance to wilt caused by the oomycete *Phytophthora capsici*. Seeds were sown in January 2012 and the commercial varieties were grafted 31 days after sowing. Five weeks after the grafting, the plants were transplanted to beds under net shading. In this study, the soil used had a loamy sandy-clayey texture (29.84% clay, 12.08% silt, and 57.36% sand), inorganic N of 50.17 ppm, P of 64.14 ppm, CIC of 32.5 me/100g, electrical conductivity of 0.84 ds/m, MO of 1.68%, and pH 7.72.

The fertilization program for a cycle of 220 days consisted of the following forms and application rates: NH_4_NO_3_ (50.4 g∙m^2^), UAN32 (37.7 g∙m^2^), 5-30-00 (N-P-K) (56 g∙m^2^), KNO_3_ (44.8 g∙m^2^), Ca (NO_3_)_2_ (162.3 g∙m^2^), K_2_SO_4_ (201.3 g∙m^2^), and MgSO_4_ (107.5 g∙m^2^), supplementing the fertilization with commercial products.

The experimental design was random block with four treatments: (1) grafted Fascinato (Fascinato/Terrano); (2) grafted Janette (Janette/Terrano); (3) ungrafted Fascinato; (4) ungrafted Janette these latter two treatments being taken as control. Also, four samples were taken over the same production cycle of the year 2012: the first on 16 July, the second on 6 August, the third on 20 August, and the fourth on 3 September. 

Harvested fruits were classified as commercial quality according to the México Supreme Quality Standard [48]. One lot from each treatment was evaluated and, from each lot, fruits were sampled from 10 plants, these being considered as the experimental unit. The fruits were cleaned and taken to the laboratory to measure the content in vitamin C, total phenols, lycopene, β-carotene, and antioxidant capacity.

### 3.2. Determination of Bioactive Compounds

#### 3.2.1. Vitamin C

The vitamin C was extracted by the method of Doner and Hicks [49]. The fresh bell pepper pulp was milled and 10 g were placed in 20 mL of an extraction mixture, which was homogenized and afterwards filtered before being analyzed by high-resolution liquid chromatography (HPLC). The filtrate was injected into a Varian HPLC ProStar 320 with a UV-Vis detector (ProStar 210) amine column, 10 m, Varian, UV-Vis lamp at 268 nm and a 20 µL injection loop.

#### 3.2.2. Total Phenols

The Folin-Ciocalteu method was used to quantify the total phenols, as in Hernández *et al.* [50], using gallic acid as a standard. The fresh bell pepper was diced into small pieces and a 100-g sample was used for three consecutive extractions with methanol (3 × 400 mL) at room temperature for 24 h. The three extracts together were evaporated to dryness and the concentrate was dissolved in methanol (5 mg/mL) and filtered through nylon membranes (MNYL type, 0.2 µm; Whatman, Kent, UK), and 20 µL of sample were injected into an HPLC Varian ProStar 320. The column used was a LiChrosphere 100 FR-18 (125 × 4 mm, 5 mm) and a diode-arrangement detector. The extract was gradient eluted with formic acid at 5% in water (A) and methanol (B) with a flow rate of 1 mL/min. The gradient program was 30% B (0–15 min), 40% B (15–20 min), 45% B (20–30 min), 60% B (30–50 min), 80% B (50–65 min), and 100% B (65–75 min). The compounds eluted were detected at 280 and 340 nm and the UV spectrum was registered from 190 to 400 nm. The spectrum generated by the diode-arrangement detector was used to identify the compounds detected. The standard was dissolved methanol (5 mg/mL) filtered through a nylon membrane (MNYL type, 0.2 µm; Whatman) prior to its injection into the HPLC.

#### 3.2.3. Lycopene

The lycopene content was quantified as in Cucu *et al.* [51]. All the steps in the preparation of the sample were performed under dim light. The fresh pepper was finely milled and 1 g of sample was mixed for 1 min with an extraction-buffer solution (hexane/acetone/ethanol 2:1:1). The mixture was filtered and deposited in a separation funnel. A saturated solution was added and mixed for one min and biphasic formation was allowed. The aqueous phase was discarded and the hexane recovered. This phase was filtered with 5 g of NaSO_4_ anhydride, which was rinsed twice with 2.5 mL of the extraction mixture. The filtrate was dried under nitrogen and the resulting residue was redissolved in tetrahydrofuran (THF) with butylated hydroxytoluene (BHT), and triethylamide (TEA) at 0.05%. The sample was injected into an HPLC Varian ProStar 320 with UV-Vis ProStar 210 detector at 472 nm using a reversed-phase C18 column of 10 cm with MeOH/isopropyl alcohol/THF (30:30:35) containing 250 ppm BHT and 0.05% TEA as the mobile phase. The flow was 1 mL/min, the temperature of the column was 35 °C, and the injection volume was 20 µL. The lycopene was identified by comparison with the retention time of a lycopene standard (90% *E*-isomers) used as a reference and the quantified by external standard calibration based on the area of the peaks. As a control for the analytic process, β-carotene (1.25 µg/mL) was added to the lycopene standard. The total lycopene content was quantified as the sum of the areas of the peaks of all the *E* and *Z* isomers, and based on the standard lycopene curve.

3.2.4. β-Carotene

The β-carotene was analyzed by the technique of Mejia *et al.* [52]. The seeds and stem were removed from the bell pepper, which was diced into small pieces, and 10 g of sample were taken in triplicate. Each sample was mixed with 1 g of magnesium carbonate, 10 g of sodium sulfate and 75 mL of THF, stabilized with 0.015% BHT. The mixture was homogenized at low speed for 2.5 to 3 min and cooled with ice for 3–5 min. This process was repeated a second time and finally filtered. The residue was mixed with a second portion of 75 mL of THF and the extraction was repeated. The two filtrates were combined and the total filtrate was evaporated using a rotatory evaporator (Buchi) at ≤40 °C. Afterwards the extract was redissolved with 25 mL of THF to be analyzed by HPLC. The analysis was made by CLAR in a chromatograph (HPLC Varian ProStar 320), with a UV-Vis ProStar 210 detector and a C18-type column (Waters Novapack) of 3.9 mm × 15 cm. The solvent system consisted of acetonitrile/THF/H_2_O (85:12.5:2.5). The analyses were made at 24 °C and detection at 460 nm. The chromatograph peaks were identified comparing the retention times with trans-α-carotene (Type V) and β-carotene (Type IV) standards and internal standards. 

#### 3.2.5. Antioxidant Capacity

The antioxidant capacity was determined using the DPPH (1,1 diphenyl-2-picryl-hydrazyl) method, following Zhuang *et al.* [4]. The stems and seeds of the bell pepper were removed and, with 5 g of fresh pepper, an extraction was made by stirring in 75 mL of 80% ethanol at room temperature for 24 h. The extract was filtered and concentrated in a rotatory evaporator at 40 °C. A reaction mixture was prepared with 2 mL of DPPH solution in 0.1 mM of methanol and 0.4 mL of extract. This mixture was incubated in darkness for 30 min and its absorbance was recorded at 517 nm in a microplate reader.

The DPPH radical-scavenging activity was calculated according to Equation (1):

% Scavenging activity = ((A_control_-A_extract_)/A_control_ ) × 100 (1)

where A_control_ = absorbance of the control, and A_extract_ = absorbance of the extract.

### 3.3. Statistical Analysis

In the present study, the effect of the variety of bell pepper, the rootstock, and the sampling date were evaluated with respect to the content in bioactive compounds and antioxidant capacity, using an analysis of variance, and a comparison of means was made by Tukey’s test [53]. The means were accepted as significantly different at a 95% confidence interval (*p* ≤ 0.05). The results were expressed as the mean ± standard deviation. In addition, an analysis was made of the correlation between the antioxidant capacity and the bioactive compounds of the four treatments evaluated. All the compounds were reported in mg per g of fresh tissue and the analyses were made in triplicate.

## 4. Conclusions

The results of this study showed differences in the content of bioactive compounds in two commercial varieties of bell pepper. The Fascinato variety had higher contents of these compounds and greater antioxidant capacity than did Janette. In addition, these characteristics are favored by grafting, indicating that Terrano is a suitable rootstock to improve the nutritive quality of the commercial Fascinato and Janette varieties of bell pepper.
